# Residual Helicity at the Active Site of the Histidine Phosphocarrier, HPr, Modulates Binding Affinity to Its Natural Partners

**DOI:** 10.3390/ijms221910805

**Published:** 2021-10-06

**Authors:** José L. Neira, David Ortega-Alarcón, Bruno Rizzuti, Martina Palomino-Schätzlein, Adrián Velázquez-Campoy, Alberto Falcó

**Affiliations:** 1 IDIBE, Universidad Miguel Hernández, 03202 Elche, Spain; alber.falco@umh.es; 2 Joint Units IQFR-CSIC-BIFI, and GBsC-CSIC-BIFI, Instituto de Biocomputación y Física de Sistemas Complejos (BIFI), Universidad de Zaragoza, 50018 Zaragoza, Spain; dortega@bifi.es (D.O.-A.); bruno.rizzuti@cnr.it (B.R.); adrianvc@unizar.es (A.V.-C.); 3 Departamento de Bioquímica y Biología Molecular y Celular, Universidad de Zaragoza, 50009 Zaragoza, Spain; 4 CNR-NANOTEC, SS Rende (CS), Department of Physics, University of Calabria, Via P. Bucci, Cubo 31 C, 87036 Rende, Italy; 5 Centro de Investigación Príncipe Felipe, Calle Eduardo Primo Yufera 3, 46022 Valencia, Spain; mpalomino@cipf.es; 6 Instituto de Investigación Sanitaria Aragón (IIS Aragón), 50009 Zaragoza, Spain; 7 Centro de Investigación Biomédica en Red en el Área Temática de Enfermedades Hepáticas y Digestivas (CIBERehd), 28029 Madrid, Spain; 8 Fundacion ARAID, Government of Aragon, 50009 Zaragoza, Spain

**Keywords:** binding, circular dichroism, peptides, isothermal titration calorimetry, NMR, fluorescence

## Abstract

The phosphoenolpyruvate-dependent phosphotransferase system (PTS) modulates the preferential use of sugars in bacteria. The first proteins in the cascade are common to all organisms (EI and HPr). The active site of HPr involves a histidine (His15) located immediately before the beginning of the first α-helix. The regulator of sigma D (Rsd) protein also binds to HPr. The region of HPr comprising residues Gly9-Ala30 (HPr^9–30^), involving the first α-helix (Ala16-Thr27) and the preceding active site loop, binds to both the N-terminal region of EI and intact Rsd. HPr^9–30^ is mainly disordered. We attempted to improve the affinity of HPr^9–30^ to both proteins by mutating its sequence to increase its helicity. We designed peptides that led to a marginally larger population in solution of the helical structure of HPr^9–30^. Molecular simulations also suggested a modest increment in the helical population of mutants, when compared to the wild-type. The mutants, however, were bound with a less favorable affinity than the wild-type to both the N-terminal of EI (EIN) or Rsd, as tested by isothermal titration calorimetry and fluorescence. Furthermore, mutants showed lower antibacterial properties against *Staphylococcus aureus* than the wild-type peptide. Therefore, we concluded that in HPr, a compromise between binding to its partners and residual structure at the active site must exist to carry out its function.

## 1. Introduction

The survival of bacteria is based on their capacity to sense variations in their environments and, as a response, to adapt their metabolic systems by turning on/off the expression of specific genes [1,2]. One of those sensory systems is the bacterial phosphoenolpyruvate (PEP) sugar-dependent one, the so-called PEP-dependent phosphotransferase system (PTS). The PTS controls the preferential use of carbon sources in bacteria [3,4]. It is involved in the transport and release of carbohydrates (PTS sugars) through the cell external membrane; furthermore, the PTS also intervenes in the movement of bacteria towards carbon sources (chemotaxis) in nitrogen metabolism and in regulation of other metabolic pathways in both Gram-negative and Gram-positive bacteria [2,5,6]. The regulation of such pathways occurs through phosphorylation of its target proteins, and by interaction between the phosphorylated proteins. The target proteins of HPr can be signal transductors, transporters, catabolic enzymes, and in many cases, transcriptional regulators [5,6,7,8]. The organization of the PTS is similar in all bacterial species described so far: it is formed by a sequence of phosphoryl-transfer steps occurring through several proteins, from PEP to the sugar-specific enzyme II permeases (EIIs). The first two proteins in the cascade are common to all PTS substrates (the so-called “general PTS proteins”): the phosphocarriers EI and HPr, where phosphorylation occurs at some specific histidine residues. The 64-kDa protomer of EI protein, in all the species described so far, exhibits a dimer–monomer equilibrium. The proteolytic cleavage of the EI of *Escherichia coli* yields two domains [9], which also have been observed in different EI from other species [9,10,11,12]. The N-terminal domain of EI, EIN, roughly comprises the first 230 residues of the intact EI: it contains the HPr-binding domain including the active-site histidine [13,14], and forms a four-helix bundle. The structure of HPr is an open-face beta-sandwich formed by three α-helices packed against a four-stranded β-sheet [15]. The active site of HPr is located close to the N-terminal end, and is formed by a single residue, His15, just immediately before the N-cap of the first α-helix. His15 undergoes a PEP-dependent phosphorylation by the action of EI at the first step of the PTS. The helix nearby His15, together with the preceding loop, is the region involved in the binding to EIN (Figure S1).

Bacteria also respond to changes by modulation of the function of their transcription machinery. Transcription in bacteria is carried out by a single multi-subunit RNA polymerase (RNAP). RNAP binds to one of the σ factors with a specific promoter recognition activity [16]. Bacterial σ factors are multidomain proteins, including different domains that recognize specific promoter elements [17]. Bacteria have several σ factors; for instance, in *E. coli* there are seven factors required for the transcription of more specialized genes [18,19]. The anti-σ factor of σ^70^ is the 158-residue-long protein Rsd (regulator of sigma D), which prevents the transcription of σ^70^-dependent promoters [20,21]. It has been shown that the dephosphorylated form of HPr in *E. coli* also binds tightly to Rsd, inhibiting the formation of the complex between Rsd and σ^70^ [22,23]. The binding region of HPr involves the same one intervening in the binding to EIN [23]. We have recently shown that the Rsd from *E. coli*, Rsd^ec^ (P0AFX4), is capable of binding HPr from *Streptomyces coelicolor*, HPr^sc^ [24]. In *S. coelicolor*, there are as many as 65 σ factors, and the interaction of any of their anti-σ factors with HPr^sc^ has not yet been identified [25].

*Streptomyces* is an actinomycete, Gram-positive organism with high content of G + C base pairs in its genome. We have carried out an extensive description of the structures and conformational stabilities of HPr^sc^ (O50515) and EI (EI^sc^) (Q9KZP1) to understand their binding in the PTS cascade [11,12,26,27]. We have also characterized the interaction of EI^sc^ with wild-type HPr^sc^ [28] and that of the phosphorylated species [29], as well as that of the N-terminal domain of EI^sc^ (EIN^sc^) with intact HPr^sc^ [10,29]. We have also shown that the peptide comprising residues Ala9-Gly30 of HPr^sc^ (HPr^9–30^) is mainly disordered in aqueous solution (with about 7% of helical population) [30]. The peptide binds to the intact EI^sc^ [28] with a *K*_d_ of 230 µM, and to EIN^sc^ with a *K*_d_ of 8 µM (both measured by isothermal titration calorimetry (ITC)) [31]. The latter value was similar to that measured for the binding reaction between intact HPr^sc^ and EIN^sc^ (12 µM [31]). In addition, Rsd binds to HPr^9–30^ with a *K*_d_ of 2.5 µM, which is also similar to that measured for the intact HPr^sc^, 2 µM [24] (again, as measured by ITC).

In the present work, we wondered whether an increase in the affinity for both EIN and Rsd could be obtained by increasing the helicity of isolated HPr^9–30^ (the peptide that contains the first α-helix of the protein, encompassing residues Ala16-Thr27). To that aim, and by gathering indications obtained by comparing sequences of different species for HPr, we mutated residues Pro18, Ile21, Phe22, and Val23 of the wild-type sequence to Ala, obtaining at the end six different peptides (including a triple- and quadruple-mutant, in addition to the wild-type HPr^9–30^). The rationale behind the mutations in the wild-type sequence was the following. Although the Pro residue is highly conserved among the HPr species, the mutant P18A was designed because proline is a well-known helix-breaker [32], and we wanted to evaluate the effect of increasing the length of the helix at its N terminus. The residue Ile21 was mutated because this position is occupied by Leu or Cys in HPr from other species, therefore variability in this position is tolerated. Similarly, the F22A mutant was designed because the preferred residue at this position is Leu or Ile in other variants. Finally, Val23 is more frequently an Ala residue in other species. These residues do not make a large number of contacts with either EIN or Rsd in the complexes of the whole HPr. Our results showed that, in all mutants, the helicity was slightly increased compared to the wild-type sequence, as measured by far-UV (ultraviolet) CD (circular dichroism). NMR in aqueous solution of the peptides also suggested a slight increase of the helicity in the peptides; moreover, in the presence of the organic solvent 2,2,2-trifluoroethanol (TFE), the helical structure of the peptides comprised the same residues as in the intact, wild-type protein (that is, Ala16-Thr27). However, the mutation of any of the above-mentioned residues yielded a lower affinity than the wild-type peptide towards both target proteins. Furthermore, antibacterial activity against *Staphylococcus aureus* decreased in all the mutants, when compared to that of the wild-type sequence. Therefore, there is a subtle balance between residual helicity of HPr and binding affinity towards both EIN and Rsd, which is altered by any of the mutations explored.

## 2. Results

### 2.1. The Peptides Were Disordered Monomers in Aqueous Solution

As a preliminary step towards the aim to assess the ability of peptides for binding to Rsd^ec^ and EIN^sc^, we firstly biophysically characterized them by using far-UV CD and NMR. The latter technique allowed us to assign the proton resonances of the peptides. Peptides were named according to their mutations compared to the wild-type sequence.

**Figure 1:**
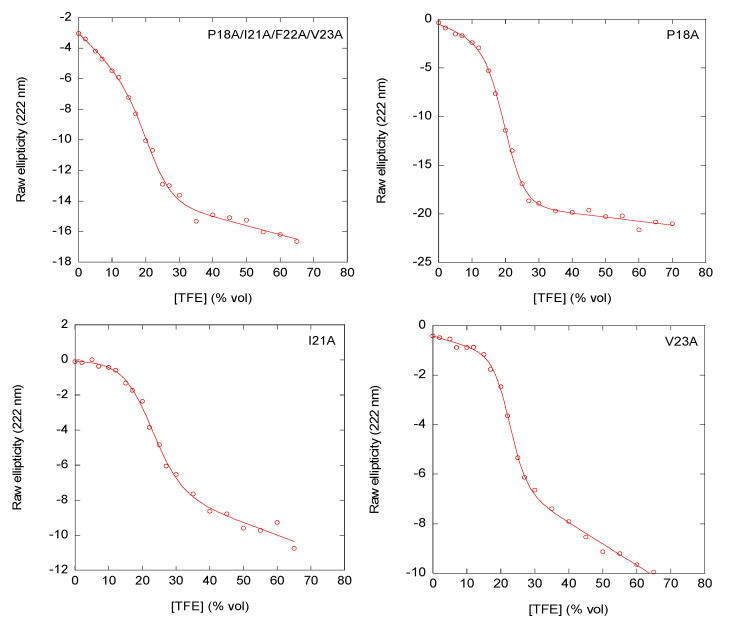
TFE titrations of selected HPr peptides followed by far-UV CD. Sigmoidal change in the [Θ] at 222 nm, [Θ]^222^, as the TFE concentration was increased for selected peptides. The line is the fitting to a two-state model [37,38].

The far-UV CD spectrum of the wild-type peptide in aqueous solution has been previously described [30]. The far-UV CD spectra of the mutants showed similar features, with an intense band at 198 nm, suggesting that the peptides were mainly disordered in aqueous solution (Figure S2) [33,34,35]. We used two different procedures to estimate the percentage of helical structure in the peptides. Firstly, from the ellipticity at 222 nm, [Θ]^222^, we could estimate the percentage of helical structure [36], assuming that the value of [Θ]^222^ for a fully formed α-helix was −39,500 deg cm^2^ dmol^−1^. The results (Table 1) indicated that, except for the I21A peptide, the mutants had a larger value of the [Θ]^222^, and therefore a larger value of helicity than the wild-type one (Table 1). This result was not entirely surprising, because the mutations introduced a larger number of residues of alanine, which is a well-known helix-inducer [32]. However, it is important to keep in mind that the findings from the molar ellipticity can be affected by the presence of aromatic residues, which also absorb at this wavelength [33,34,35]. Secondly, TFE titrations followed by CD were employed to estimate the equilibrium constant (*K*) for the disordered ↔ helical transition reaction [37,38], assuming that there were no intermediates (as suggested by the presence of an isodichroic wavelength for each peptide titration). Unlike the above analyses on helicity, which made assumptions about how the ellipticity signal at 222 nm related to secondary structure content, TFE titration allowed the determination of helical content derived from the free-energy change of such equilibrium (disordered ↔ helical). The [Θ]^222^ increased in absolute value for all peptides as TFE concentration was raised (Figure 1). The slopes of the titration curves at the beginning and the end of the sigmoidal transitions indicated that the population of disordered peptide confirmations (low TFE concentrations) or folded conformations (high TFE concentrations) was changing, although in a non-cooperative manner, as the concentration of co-solvent was varied. The titrations for all the peptides showed a similar cooperativity, with *m*-values ranging from 150 to 220 cal/(mol% (*v*/*v*)) (Table 1); although for the peptide I21A, the *m*-value was slightly lower (130 cal/(mol% (*v*/*v*))). The large value of this parameter found for all the peptides indicated that the transition (disordered ↔ helical) was very cooperative. On the other hand, the [TFE]_1/2_-value (the midpoint of the transition is given in % (*v*/*v*)) did not differ significantly among all the peptides, ranging from 19 to 24% (*v*/*v*) (Table 1, Figure 1). These values yielded a similar free energy for the disordered ↔helical reaction for most of the peptides (except for I21A), and therefore a low population (lower than 1% in all peptides) of helical structures, in contrast to what was obtained from the value of the molar ellipticity at 222 nm, where an increase in the content of alanine residues resulted in a larger helicity. Furthermore, not only the absolute values of the helical populations were lower than those obtained from the value of [Θ]^222^, but hose with a lower helical population were I21A and V23A (that is, those with the largest free-energy value for the disordered ↔ helical reaction) (Table 1). It is interesting to note here that similar *m*- and [TFE]_1/2_-values, within the error, were previously obtained for the titration of the wild-type peptide under slightly different conditions (in phosphate buffer, pH 7.0, 10 mM) [30]. 

We also carried out MD simulations of the HPr peptides to obtain further insight into their helical propensity. Helical structure is crucial for proteins in general, therefore simulation force fields are parameterized to reproduce it at best, although its occurrence may be overestimated [39]. More importantly, it critically depends on the water model used, especially for disordered protein regions. In spite of such complications, simulations can provide an approximate ranking of the helical tendency for the peptides and, more indirectly, to their ordered/disordered propensity. Table 2 reports the percentage of helical structure calculated for our HPr fragments by using different water models. The most evident finding was that the sole simulation runs with the water model TIP4P-D were capable of predicting a larger amount of helical structures for the triple- and quadruple-alanine mutants, in agreement with our findings when reporting only the molar ellipticity; furthermore, the P18A peptide was correctly assessed as the one with the larger helicity among the single-point mutants (in agreement with the findings from the measurements of molar ellipticity at 222 nm; Table 1). These observations were particularly interesting because the model TIP4P-D was developed to better reproduce the propensity towards disordered protein states over ordered ones [40], thus it normally tends to decrease rather than increase the amount of secondary structure. Therefore, the results suggest a tendency towards a very disordered (and likely context-dependent) structure of the HPr peptides, and especially those with multiple alanine mutations. It is also worth noting that, in all cases, the region involving helical fold was the one expected in the structure of the intact, wild-type HPr (residues Ala16-Thr27).

To sum up, from far-UV CD experiments and MD simulations, it could be concluded that the peptides were mainly disordered in aqueous solution, and the mutations did not seem to substantially increase the amount of helical structures, when compared to the wild-type one, although they were designed to increase such population by introducing additional alanine residues. The values of the residual helicity in the peptides varied among the far-UV CD approaches used, as well as in the comparison between the in silico and experimental methods. Then, it could be deduced that the residual helical structure was highly flexible.

**Figure 2:**
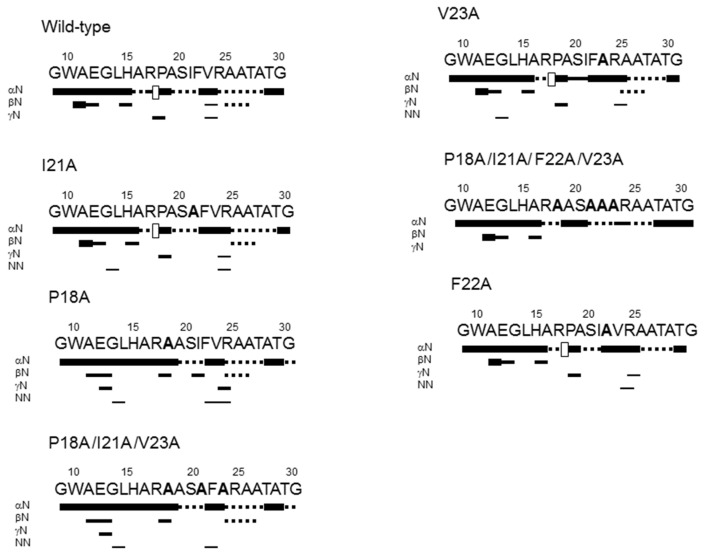
**Conformational characterization of the HPr peptides by NMR.** An NOE diagram of isolated HPr peptides in aqueous solution. NOEs were classified into strong, medium, or weak, as represented by the height of the bar underneath the sequence; signal intensity was judged by visual inspection from the NOESY experiments. The dotted lines indicate NOE contacts that could not be unambiguously assigned. The blank squares correspond to the sequential αδ(*i*, *i* + 1) NOE observed between a residue preceding a proline and the proline itself.

To further confirm the inherently disordered nature of the HPr peptides, we carried out homonuclear 2D-^1^H-NMR experiments. We observed that the peptides were mainly disordered in aqueous solution, as shown by the absence of long- or medium-range NOEs along the chain (Figure 2); some sequential NN(*i*, *i* + 1) NOEs (and ROEs) were observed for particular residues of the mutants, around the site where the mutation was introduced. Assignments of the triple and quadruple mutants in aqueous solution or 40% TFE (see below) were especially difficult due to the overlapping of several alanine resonances.

We also carried out NMR experiments in the presence of 40% TFE, when the titration of all peptides had reached a plateau (Figure 1). We conducted these experiments to ensure that all the peptides had a similar tendency to adopt a helix-like conformation for the same residues. Both the NOE pattern (presence of NN(*i*, *i* + 1), NN(*i*, *i* + 2), NN(*i*, *i* + 3), αN(*i*, *i* + 3), αN(*i*, *i* + 4), and αβ(*i*, *i* + 3) contacts) (Figure S3) and the upfield shift of the H_α_ protons indicated the presence of helixlike conformations between Ala16 and Thr26 (see Supplementary tables); these residues were also part of the first α-helix in the intact HPr. Then, there was an intrinsic tendency in that region to acquire a helix-like structure, even when mutations were made.

We also determined whether the peptides in aqueous solution were monomeric or had a tendency to oligomerize, and to that end, we conducted diffusion-ordered spectroscopy (DOSY) experiments (Table 3). All the peptides showed a translational diffusion coefficient, *D*, in agreement with the value expected for a hydrodynamic radius, *R*_h_, of a monomeric polypeptide chain of the corresponding molecular weight. Therefore, the peptides were monomeric in aqueous solution under our experimental conditions. It is interesting to note that for the wild-type peptide, we previously determined *D* at infinite dilution [30], and the value obtained (1.4 ± 0.4 cm^2^ s^−1^) agreed with that measured in this work by using dioxane as reference.

### 2.2. Binding of the HPr Peptides to EIN^sc^ and Rsd^ec^

By following a two-part approach, we tested whether the peptides were capable of binding to any of the two proteins that intact HPr^sc^ binds [25,29,31], and whether the binding affinity was modified by the introduced mutations. First, we used steady-state fluorescence and far-UV CD to determine whether there were some spectroscopic changes in the signal of the proteins and/or the peptides upon binding. Second, we used fluorescence spectroscopy and ITC to determine the affinity constant in the association between the peptides and each of the proteins.

**Figure 3:**
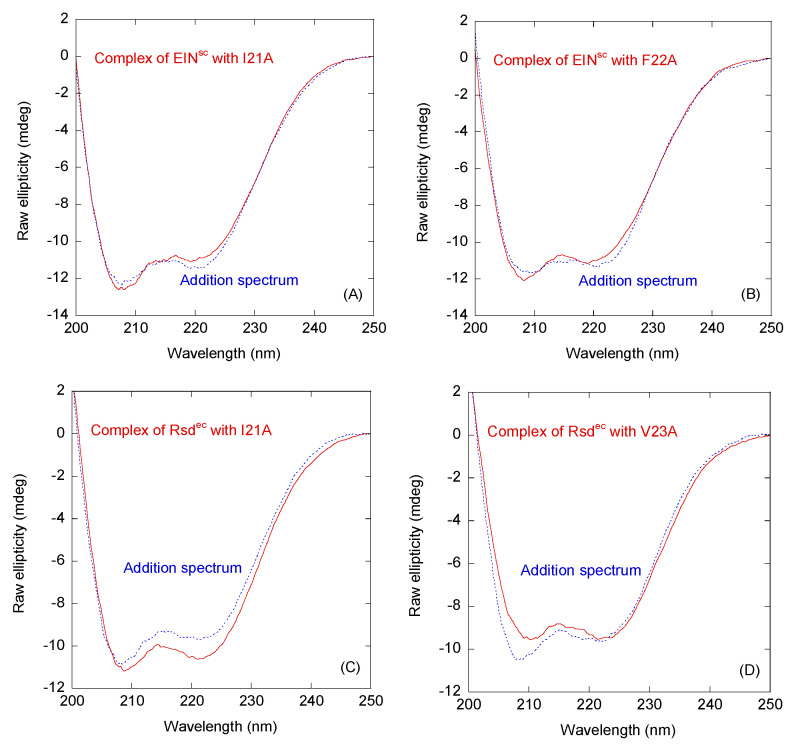
Binding between two selected HPr peptides and EIN^sc^ and Rsd^ec^, as monitored by spectroscopic techniques. (**A**) Far-UV CD spectrum of the complex EIN^sc^/I21A and the one obtained by the addition of the spectra of the two isolated polypeptide chains. (**B**) Far-UV CD spectrum of the complex EIN^sc^/F22A and that obtained by the addition of the spectra of the two isolated polypeptide chains. (**C**) Far-UV CD spectrum of the complex Rsd^ec^/I21A and that obtained by the addition of the spectra of the two isolated polypeptide chains. (**D**) Far-UV CD spectrum of the complex Rsd^ec^/V23A and the one obtained by the addition of the spectra of the two isolated polypeptide chains. The experiments were carried out at 25 °C in 50 mM Tris buffer (pH 7.0).

A comparison of the far-UV CD spectra of the complexes with the addition ones, obtained by the sum of the spectra of the corresponding isolated polypeptides (Figure 3), indicated that the variations between the spectra were not very large for both Rsd^ec^ and EIN^sc^. These findings indicated that the binding did not alter the structural properties of the protein or the peptides (Figure 3) or, alternatively, that the binding was spectroscopically silent from the point of view of monitoring it by using far-UV CD spectroscopy. The changes in the fluorescence spectra were not very large either; nevertheless, small changes were observed, and these variations could be useful in determining the affinity by fluorescence titrations (see below). Therefore, from the fluorescence measurements, it was evident that the binding took place, and that the environment around the sole tryptophan of the peptides changed in the presence of the other proteins.

**Figure 4:**
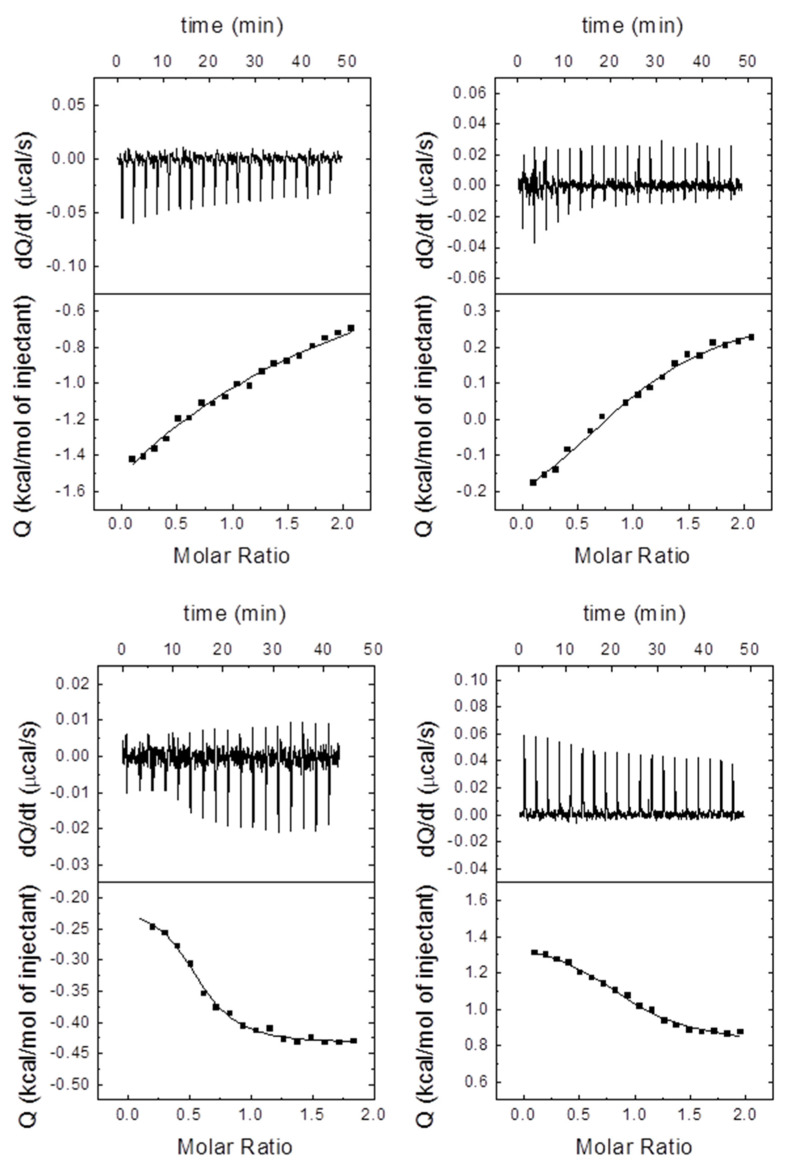
Binding between two selected HPr peptides and EIN^sc^ and Rsd^ec^, as monitored by ITC. Interaction of V32A with EIN^sc^ (top left) and Rsd^ec^ (top right), and P18A/I21A/F22A/V23A with EIN^sc^ (bottom left) and Rsd^ec^ (bottom right). The upper plots show the thermogram (thermal power required to maintain a minimal temperature difference between sample and reference cells as a function of time), and the lower plots show the binding isotherm (titrant-normalized heat effects per injection as a function of the molar ratio, the quotient between the titrant and titrand concentrations in the sample cell). The continuous lines are the fitting curves to a single binding-site model. The experiments were carried out at 25 °C in 50 mM Tris buffer (pH 7.0).

To quantify the extent of such binding, we carried out fluorescence titrations and ITC measurements. In the case of EIN^sc^, we could not obtain a measurable value for most of the mutants, either by ITC or fluorescence (Table 4, Figure 4 and Figure S4). Therefore, it seemed that the mutations of P18A, I21A, F22A or V23A essentially hampered the binding to EIN^sc^. In addition, there was not a direct relationship between the number of mutations made and the changes in the affinity; for instance, the affinity of the quadruple mutant for EIN^sc^ was two-fold that of the wild-type peptide. In the few cases that could be measured for EIN^sc^, the mutant with the lowest affinity (i.e., larger *K*_d_) was V23A. On the other hand, for Rsd^ec^, we were able to determine the affinity of all the peptides by ITC (although we could not do so by using fluorescence). We observed that any mutation increased the dissociation constant, and then decreased the affinity. Therefore, a larger number of mutations does not imply a larger *K*_d_, and there must be compensatory effects (structural versus interaction effects) in the mutations. Finally, it is important to indicate that, although the absolute values of the dissociation constants obtained from fluorescence and calorimetric were not exactly the same, the changes induced by the mutations in the HPr-peptides were similar.

Interpretation of the enthalpic and entropic contributions for the binding of the different peptides was questionable. Experiments were performed in Tris, a buffer with a large ionization enthalpy (ca. +11 kcal/mol), and even for a small net number of protons exchanged upon complex formation, the contribution of the buffer to the observed enthalpy (and entropy) was non-negligible. In addition, different peptides might exchange a different net number of protons, making comparisons difficult and unsound. Even if non-ionizable residues were mutated to alanine, those substitutions might alter the ionization properties of ionizable side-chains and the alterations exerted upon complex formation. However, that was not a problem for the affinity, and dissociation constants were reliable, because the buffer did not affect the binding affinity as long as the p*K*_a_ of the buffer was close to the experimental pH, and could be compared, which was the purpose of using ITC for studying these interactions.

### 2.3. The Fragments Displayed Antibacterial Activity against Staphylococcus aureus 

To ascertain whether the mutated positions within the HPr peptides also had an effect on the capacity to inhibit the growth of *S. aureus*, their minimal inhibitory concentrations (MICs) were determined by the two-fold broth microdilution method (Table 5). Thus, it was revealed that I21A, V23A, and P18A/I21A/F22A/V23A could not inhibit the bacteria growth even at the highest peptide concentration tested (400 µM). Among the peptides showing at least a minimum antibacterial activity, F22A was the least potent one, showing an MIC of 400 µM. The peptides endowed with the highest antibacterial activity were, in ascending order, wild-type, P18A/I21A/V23A, and P18A, with MICs of 40, 60, and 120 µM, respectively. Thus, it seemed that the mutation F22A had a huge effect on MIC (either in the isolated mutation or in the quadruple mutant), and the triple mutant most likely had a low MIC because Phe22 was not mutated. Phe22 is a key residue to attain a good antibacterial effect.

## 3. Discussion

The dissociation constant of the interaction between Rsd and HPr in *E. coli* reported as 8.87 nM [22], as measured by surface plasmon resonance (SPR). Our measurements, by using fluorescence and biolayer interferometry (BLI) for the intact HPr^sc^ [24], suggested that the binding occurred in the low micromolar range (0.2 to 1.5 µM, depending on the technique), in agreement with previous measurements [22,23]. We previously showed that the peptide comprising residues Gly9-Ala30 of HPr^sc^ also binds to Rsd^ec^ with an affinity similar to that of the intact protein [24]. In this work, we showed that mutations of residues Pro18, Ile21, Phe22, and Val23 into alanine in such peptide led to a decreased affinity for Rsd^ec^, but such decrease did not seem to have a clear direct relationship with the number of mutations, or with the position where they were located. The changes were more drastic in the case of EIN^sc^, because we could measure an affinity constant only for a few peptides. These results seemed to suggest that the first helix of HPr was optimized for binding with EIN^sc^ rather than with Rsd^ec^. All residues considered were involved in attaining the more favourable binding (i.e., the lowest dissociation constant), and an increase in helicity yields to a lower *K*_d_. That is, in broad terms, residual helicity played a role in defining binding affinity. These findings suggested that disorder and residual helicity of HPr^sc^ at its first helical region had a key role in determining the balance between binding affinities and binding specificities in the PTS system. In principle, increasing helical content could be considered beneficial for the interaction, because: (1) the HPr region interacting with EI^sc^ or Rsd^ec^ adopted an α-helical conformation; (2) the wild-type HPr peptide was disordered in solution; and (3) the binding resulted in the formation of an α-helix in the peptide. However, the mutations introduced could affect the intramolecular interactions, and thus increase the dissociation constant for the mutant peptides. In addition, the α-helix propensity of that region might be not optimal in wild-type HPr (although optimal for interacting with a preferred target, e.g., EI), but the region still adopted an α-helical conformation within full-length HPr because of the structural constraints and the network of cooperative intramolecular interactions, absent in the isolated HPr peptide.

It could be argued that we did not find any direct evidence that the peptides did not acquire a helical structure upon binding [14]; however, since the region encompassing the peptides was folded in the complexes between the intact proteins, it does not seem unreasonable to assume that there was a folding-upon-binding event when the peptides and EIN and Rsd were used. In addition, we recently obtained a fragment comprising the first 48 residues of HPr, and we observed that, upon binding to EIN and Rsd, the residues that showed the largest variations in chemical shifts or broadenings were those around His15 and Arg17 [44], suggesting that the conformation around this region changed in the interaction with such large proteins.

It is important to indicate that the small-scale alanine scanning carried out in this work with the peptides consisted of removing residues (such as Phe22 and Val23) that did not have a large number of interactions with EIN or Rsd, and therefore in addition to attempting to increase helicity, we could also be removing van der Waals interactions and side-chain–side-chain contacts with nearby residues of the peptide. In fact, according to the structure of the complex between EIN and HPr from *E. coli* (PDB entry 3EZE) the residues of HPr that have a larger number of contacts with EIN are Thr16, Arg17, and Ala20 (or Ala16, Arg17, and Ser20 in *S. coelicolor*; Figure 2) [14], and in the residues mutated in the peptide, only Ph22 and Val23 showed hydrophobic contacts with an α-helix in EIN (PDB entry: 3EZE) [14]. Furthermore, in the case of Pro18 to alanine, the mutation could affect the helix-capping, and it could potentially change the entropy of binding. However, keeping in mind those limitations of our approach, alanine scanning (with a wider range of mutations than those used in this work) has been used to study the relationship between residual helicity in a protein and the binding to other partners (either in homo-dimerization or hetero-dimerization experiments) [45,46].

Along these lines, as observed in the antibacterial assays performed, the peptides that showed the lowest dissociation constants here (i.e., 1.4, 2.2, and 2.6 µM for wild-type, P18A, and P18A/I21A/V23A, respectively) (Table 4) were also those that achieved the lowest MICs (i.e., 40, 60, and 120 µM for wild-type, P18A/I21A/V23A, and P18A, respectively) (Table 5). Therefore, roughly, the higher the binding affinity of the HPr peptides for Rsd^ec^, the higher the inhibition of the bacterial growth, although it should be considered that some other factors might be hampering the activity of the peptides in biological systems, such as their penetration ability into bacteria. It should also be noted that the designed peptides presented antibacterial activity against a species of bacteria (*S. aureus*) different from those initially used for peptide design (*Streptomyces coelicolor*). Although the MICs obtained were too high to suggest their use as antibacterial in their current form, the observed inhibitory effect demonstrated the circumvention of the defences of Gram-positive bacteria against antibiotics. Thus, the biophysical aspects of our investigation have direct consequences for possible biotechnological applications, since bacterial pathogens are becoming increasingly resistant to the antibiotics used to tackle infectious diseases. More than 150 antibiotics belonging to at least 17 different classes are now available, and each class operates at a specific site within the cell. However, the increasing occurrence of antibiotic resistance makes it more compelling than ever to pursue the search for new pharmacological targets [47], such as the peptides explored in this work.

## 4. Materials and Methods

### 4.1. Materials

Ampicillin, isopropyl-β-D-1-thiogalactopyranoside, deuterium oxide, and deuterated TFE were obtained from Apollo Scientific (Stockport, UK). Imidazole, Trizma base, SIGMAFAST protease tablets, sodium trimethylsilyl [2,2,3,3-^2^H_4_] propionate (TSP), and 2,2,2-trifluoethanol were purchased from Sigma-Aldrich (Madrid, Spain). Both the Luria-Bertani (LB) and Mueller-Hinton (MH) bacterial culture broths were also from Sigma-Aldrich. Amicon centrifugal devices with a cut-off molecular weight of 3.5 or 10 kDa were from Millipore (Barcelona, Spain). Dialysis tubing with a cut-off molecular weight of 3.5 kDa was from Spectrapore (VWR, Barcelona, Spain). Standard suppliers were used for all other chemicals. Water was deionized and purified on a Millipore system. The rest of the materials used were of analytical grade. 

### 4.2. Peptides

Peptides were amidated at their C-termini and acetylated at the N-termini. They were obtained from NZYTech (Lisbon, Portugal) with purity higher than 95% as tested by mass spectra. Peptide concentrations were determined from the absorbance of individual amino acids at 280 nm [48]. In addition to the wild-type sequence HPr^9–30^, the single mutants P18A, I21A, F22A, and V23A were studied, together with the triple-mutant P18A/I21A/V23A and the quadruple-mutant P18A/I21A/F22A/V23A. It is important to emphasize that the residues of HPr in all the chosen positions did not show a large number of contacts with the EIN interface [14], and the fact that they showed a larger *K*_d_ than the wild-type (Table 4) suggested that binding was not only affected by residues involved directly in the interface. Only residues Ph22 and Val23 showed hydrophobic contacts with an α-helix in EIN (PDB entry: 3EZE) [14].

We preferred using the isolated mutant peptides to study the effect of helicity on binding, instead of the entire mutated HPr, since we showed previously that the isolated wild-type peptide of HPr (Table 3) had a similar affinity for EIN and Rsd to that of the intact HPr for the same proteins [24,28,31]. By using the designed peptides (Table 1), we avoided any other possible interactions that could have the mutated residues with the rest of HPr.

### 4.3. Protein Expression and Purification 

The wild-type Rsd vector, containing the Rsd^ec^ with a His-tag, was a kind gift from Ann Hochschild (Harvard, MA, USA). The protein was expressed in the C41 *E. coli* strain [49], and the cells were grown in LB broth. The protein was purified as described [24,50]. EIN^sc^ was also expressed and purified as described [10,29,31]. Protein concentrations were determined in all cases from absorbance, and by employing the extinction coefficient at 280 nm calculated from their sequence [48].

### 4.4. Fluorescence

Regarding the presence of amino acid residues yielding a fluorescence signal, Rsd^ec^ had two Trp and eight Tyr residues, and EIN^sc^ had four Tyr residues, whereas the peptides only contained Trp10 (Table 3). Spectra were collected at 25 °C on a Cary spectrofluorometer (Agilent, Madrid, Spain), with a Peltier temperature controller. The samples were prepared the day before and left overnight at 5 °C. Before measurements, the samples were incubated for 1 h at 25 °C. A quartz cell with a 1 cm path length (Hellma, Kruibeke, Belgium) was used. In all the experiments, the slit widths were equal to 5 nm for both the excitation and emission lights. Protein samples were excited at either 280 or 295 nm. The experiments were performed between 300 and 400 nm. The signal was acquired for 1 s, and the increment of wavelength was set to 1 nm. Appropriate blank corrections were made in all spectra. For the binding experiments, 3 µM of either protein (Rsd^ec^ or EIN^sc^) and 15 µM of the corresponding peptide were mixed. The experiments in aqueous solution were acquired in Tris buffer (50 mM, pH 7.0).

For the titration between either of the two proteins (Rsd^ec^ or EIN^sc^) and the corresponding peptide, increasing amounts of the latter, in a concentration range of 0 to 30 µM, were added to a solution with a fixed concentration of each protein (3 µM). The experimental set-up was identical to that described above, including preparation of the samples the day before and their storage overnight at 5 °C. Before measurements, the samples were incubated for 1 h at 25 °C. The fluorescence of a blank solution containing only the corresponding peptide was subtracted for each data point. The dissociation constant of the complex, *K*_d_, was calculated by fitting the plot of the observed fluorescence variation to [51,52]:

(1) $$F=F_{0}+\frac{\Delta F_{\max}}{2{\left[\mathrm{Rsd}/\mathrm{EIN}\right]}_{T}}\left[\left({\left[\mathrm{Peptide}\right]}_{T}+{\left[\mathrm{Rsd}/\mathrm{EIN}\right]}_{T}+K_{d}\right)-{\left({\left({\left[\mathrm{Peptide}\right]}_{T}+{\left[\mathrm{Rsd}/\mathrm{EIN}\right]}_{T}+K_{d}\right)}^{2}-4{\left[\mathrm{Peptide}\right]}_{T}{\left[\mathrm{Rsd}/\mathrm{EIN}\right]}_{T}\right)}^{1/2}\right]$$

where *F* is the measured fluorescence at any particular concentration of the corresponding peptide after subtraction of the blank; ∆*F*_max_ is the maximal change in the fluorescence of each protein when all of the corresponding peptide was forming the complex, compared to the fluorescence of each isolated protein; *F*_0_ is the fluorescence intensity when no other protein was added; [Rsd/EIN]_T_ is the constant, total concentration of each protein (3 µM); and [Peptide]_T_ is that of the corresponding peptide, which was varied during the titration. The *K*_d_ was determined by following the fluorescence at selected wavelengths and fitting experimental data to Equation (1). Titration of the peptides with each protein was repeated three times, and in all cases, the variations among the different repetitions were less than 5%. At all used concentrations, the fluorescence was corrected by the inner-filter effects [53].

### 4.5. Circular Dichroism

Spectra were collected on a Jasco J810 spectropolarimeter (Jasco, Tokyo, Japan) interfaced with a Peltier unit. Far-UV measurements were performed with samples placed in quartz cells with a 0.1 cm path length (Hellma, Kruibeke, Belgium), with a response time of 2 s, a band width of 1 nm, and a scan velocity of 50 nm/min. For all peptides, a wide range of concentrations (10–50 µM) was used to determine whether either the shape or the intensity of the spectra was concentration-dependent. None of those features changed in the concentration range explored. The experiments in aqueous solution were acquired at 5 °C in Tris buffer (50 mM, pH 7.0). Raw ellipticity was converted to molar ellipticity, [Θ], from which the fraction of helical populations was determined as previously described [54]. For the experiments in the presence of each protein (repeated three times), the same concentration used in the fluorescence experiments was used, and the experimental set-up was the same as described above for the isolated peptides.

For experiments in the presence of TFE, the same set of experimental parameters was used. Cosolvent concentrations were indicated in percentage of volume (%). The helical population for each soluble peptide in aqueous solution was determined assuming a two-state equilibrium for the helical ↔ random-coil transition (as suggested by the presence of an isodichroic wavelength in the titrations of all peptides examined) [37,38]. The peptide concentration for these experiments was 20 µM.

### 4.6. NMR Spectroscopy

The NMR experiments were performed at 10 °C with a Bruker Avance II 500 spectrometer (Bruker GmbH, Karlsruhe, Germany) equipped with a triple resonance probe and z-pulse field gradients. The temperature of the probe was calibrated with methanol [55]. All experiments were carried out at pH 7.2 with 50 mM Tris buffer (not corrected for isotope effects) in H_2_O/D_2_O (90%/10%), unless otherwise indicated.

(a) 1D-^1^H-NMR spectra: For peptides, 1 K scans were acquired with 32 K acquisition points at concentrations ranging between 70 and 120 µM. Homonuclear 1D-^1^H-NMR spectra were processed with TopSpin 2.1 (Bruker GmbH, Karlsruhe, Germany) after zero-filling the spectra. All spectra were referenced to external TSP, considering the pH-dependence of its signals [55].

(b) Translational diffusion *NMR (DOSY):* Peptide concentrations were the same as those used in the 1D-^1^H-NMR spectra. Translational self-diffusion measurements were performed with the pulsed-gradient spin-echo sequence in the presence of 100% D_2_O. The following relationship existed between the translational self-diffusion coefficient, *D*, and the delays used during experiment acquisition [54]:

(2) $$\frac{I}{I_{0}}=-exp\left(D\gamma_{H}^{2}\delta^{2}G^{2}\left(\Delta -\frac{\delta }{3}-\frac{\tau }{2}\right)\right)$$

where *I* is the peak intensity of a particular resonance (or a group of them) at any gradient strength; *I*_0_ is the maximum peak intensity of the same resonance(s) at the smallest gradient strength (that is, at 2% of the total power of the gradient coil); *D* is the translational self-diffusion constant (in cm^2^ s^−1^); δ is the duration (in s) of the gradient; *G* is the gradient strength (in T cm^−1^); ∆ is the time (in s) between the gradients; γ_H_ is the gyromagnetic constant of the proton (in rad s^−1^ T^−1^); and τ is the recovery delay between the bipolar gradients (100 µs in our experiments). The gradient strength (*G*) was varied in 16 lineal steps, between 2 and 95% of the total power of the gradient coil of the probe. The gradient strength was calibrated by using the value of *D* for the residual proton water line in a sample containing 100% D_2_O placed in a 5 mm tube [42]. Data were plotted as *I*/*I*_0_ versus *G*^2^, and the exponential factor of the curve was:

(3) $$D\gamma_{H}^{2}\delta^{2}\left(\Delta -\frac{\delta }{3}-\frac{\tau }{2}\right)$$

from which *D* can be obtained. The duration of the gradient (δ) was 2.25 ms, and the time between the two gradients (∆) was set to 200 ms. The signal from the methyl groups between 1.1 and 0.70 ppm were used for integration of the intensity. A final concentration of 1% of dioxane was added to each peptide; the hydrodynamic radius, *R*_h_, of each peptide was obtained for comparison by assuming that the *R*_h_ of dioxane was 2.12 Å [42]. The DOSY experiment was repeated twice with fresh samples for each peptide.

(c) 2D-^1^H-NMR spectroscopy: Two-dimensional experiments were performed with a spectral width of 7801.69 Hz in each dimension, and in phase-sensitive mode by using the time-proportional phase-incrementation technique (TPPI) [56]; for all peptides, the final concentration in aqueous solution was in the range of 1.5–2 mM. Experiments were also carried out in 40% deuterated TFE at the same pH and temperature.

Standard NMR experiments, as well as TOCSY (two mixing times, 60 and 80 ms), ROESY (two mixing times, 200 and 300 ms), and NOESY experiments (two mixing times, 200 and 300 ms) were performed. Data from the TOCSY, ROESY, and NOESY experiments were acquired with a data matrix size of 4K (*t*_2_) × 512 (*t*_1_), with the MLEV17 spin-lock sequence [57] in the TOCSY experiments, and 1 s of relaxation time in all experiments. Typically, 80 scans were acquired per *t*_1_ increment, and the residual water signal was removed by using the WATERGATE sequence [58]. NOESY and ROESY spectra [59,60] typically were collected with 128 scans per *t*_1_ increment, with the residual water signal removed by the WATERGATE sequence and a relaxation time of 1 s. Data were zero-filled and resolution-enhanced with phase-shifted sine bell (DQF-COSY) or square sine-bell window functions (TOCSY, NOESY, and ROESY) optimized in each spectrum, baseline-corrected, and processed with the Bruker XWINNMR software. The ^1^H NMR resonances were assigned using standard sequential assignment processes [61] when possible (see the Results section). The random-coil chemical shift values of H_α_ protons in aqueous and TFE solutions were obtained from tabulated data for model peptides, and corrected by neighbouring residue effects [62].

### 4.7. Isothermal Titration Calorimetry (ITC) 

The experimental set-up and data processing of ITC experiments has been described previously [29,31,63]. Rsd^ec^ or EIN^sc^ (at 10–20 µM) were injected into the cell, and the peptides in the syringe (100–200 µM) in 50 mM of Tris buffer, pH 7.0. The temperature for all the experiments was 25 °C. Typically, each calorimetric titration consisted of performing 19 injections with 2 μL volume, with a time spacing of 150 s, while stirring at 750 rpm in an Auto-iTC200 high-sensitivity microcalorimeter (MicroCal, Malvern-Panalytical, Malvern, UK). The results were analysed by applying a model that considered a single binding site (assuming a 1:1 stoichiometry) for the peptide/EIN^sc^ or peptide/Rsd^ec^ interaction using a nonlinear least-squares regression analysis in Origin 7.0 (OriginLab, Northampton, MA, USA) with user-defined fitting routines.

### 4.8. Molecular Dynamics

We carried out molecular dynamics (MD) simulations to assess the tendency to possess helical structure for each of the peptides, following a protocol previously described [64,65]. The simulations were performed by using the GROMACS package with the Amber 99SB-ILDN force field [66]. Three different water models were tested: the traditional three-point model TIP3P; the more accurate four-point model TIP4P [41]; and its variant TIP4P-D [40], which precluded potential overcompaction of disordered protein states. The structures used as starting points of the simulations were built from the corresponding protein fragment in helical conformation as found in the Protein Data Bank (PDB) entry 1RZR [67].

The peptides were modelled with cappings, protonated according to a neutral pH, and solvated in a dodecahedral box with a minimum distance of 1.5 nm from any edge, and then counterions were added to obtain an overall neutral system. Simulation conditions that included the treatment of Coulombic and van der Waals interactions, use of constraints and time steps in the integration of the equations of motion, and the reference values and coupling times for maintaining constant temperature and pressure, were all as previously described [68,69]. Production runs were performed in the isobaric–isothermal ensemble for 50 ns.

### 4.9. Antibacterial Assays

The CECT59 strain of the Gram-positive bacteria *Staphylococcus aureus* from the Spanish Type Culture Collection (Colección Española de Cultivos Tipo, CECT, Universitat de Valencia, Spain) was used for determining its sensitivity to the HPr peptides studied in this work. The MIC for each peptide was determined by the two-fold broth microdilution method according to the Clinical and Laboratory Standards Institute (CLSI) guidelines [70], with some modifications as previously described [71]. For this testing procedure, a bacteria colony previously grown in MH agar plates was isolated and incubated in MH broth at 37 °C overnight to prepare the bacteria inoculum to be used in each assay. Then, the bacteria suspensions were adjusted to a 100× dilution of an 0.5 McFarland turbidity standard in MH broth and incubated with different concentrations of each peptide in round-bottom 96-well polystyrene plates (Deltalab S.L., Rubí, Spain) (50 μL/well) for 24 h at 37 °C. The peptide concentrations employed comprised two-fold dilutions starting at either 120, 160, or 400 µM, depending on the experiment. The MIC was determined as the lowest concentration of peptide that visibly inhibited bacterial growth. All assays were performed in triplicate.

## Figures and Tables

**Table 1:** Helical populations of HPr^9–30^ peptides as measured by far-UV CD.

Peptide ^a^	Helicity (%) ^a^	[TFE]_1/2_ (% (*v*/*v*)) ^b^	*m* cal/mol (% (*v*/*v*)) ^b^	∆*G*^water^ (kcal/mol) ^c^
G^9^WAEGLHARPASIFVRAATATG (wild-type)	1.6 (−612.44)	23.2 ± 0.5	166 ± 23	3.8 ± 0.5 (0.10%)
G^9^WAEGLHAR**A**ASIFVRAATATG (P18A)	2.9 (−1135.47)	19.8 ± 0.4	181 ± 17	3.6 ± 0.3 (0.15%)
G^9^WAEGLHARPASI**A**VRAATATG (F22A)	0.2 (−84.95)	22 ± 1	134 ± 27	2.9 ± 0.6 (0.5%)
G^9^WAEGLHARPAS**A**FVRAATATG (I21A)	1.7 (−681.95)	23.8 ± 0.4	197 ± 20	4.7 ± 0.5 (0.02%)
G^9^WAEGLHARPASIF**A**RAATATG (V23A)	2.5 (−988.51)	22.7 ± 0.3	217 ± 23	4.9 ± 0.5 (0.01%)
G^9^WAEGLHAR**A**AS**A**F**A**RAATATG (P18A/I21A/V23A)	6.8 (−2698.61)	19.0 ± 0.9	184 ± 32	3.5 ± 0.6 (0.17%)
G^9^WAEGLHAR**A**AS**AAA**RAATATG (P18A/I21A/F22A/V23A)	18.4 (−7305.73)	21 ± 1	157 ± 26	3.3 ± 0.6 (0.24%)

^a^ Obtained at 5 °C from the value of the [Θ]^222^, which is indicated within parentheses, and assuming that a fully formed α-helix had a [Θ]^222^ of −39,500 deg cm^2^ dmol^−1^. ^b^ Obtained from the fitting of the TFE titration curves to a two-state equation [37,38]. Reported uncertainties are fitting errors to such equation. ^c^ Calculated from the product of the *m*- and [TFE]_1/2_ values. Uncertainties in the Gibbs energy were obtained from error propagation. The values within the parentheses indicate the percentage of helical population, assuming a two-state conformational equilibrium.

**Table 2:** Helical populations of HPr^9–30^ peptides as measured by MD simulations with different water models.

Peptide	TIP3P ^a^	TIP4P ^b^	TIP4P-D ^c^
Wild-type	18%	21%	15%
P18A	12%	18%	34%
I21A	25%	34%	20%
F22A	34%	29%	20%
V23A	20%	23%	21%
P18A/I21A/V23A	33%	17%	36%
P18A/I21A/F22A/V23A	14%	14%	38%

^a^ [41]; ^b^ [41]; ^c^ [40].

**Table 3:** The sequence of the HPr^9–30^ peptides.

Peptide ^a^	MW (Da)	*D* (cm^2^ s^−1^) × 10^6^ (*R*_h_, Å) ^b^	*R*_h_ (Å) ^c^
Wild-type	2280.59	1.95 ± 0.03 (11 ± 1)	12.9
P18A	2254.51	2.0 ± 0.1 (11 ± 1)	12.8
F22A	2204.50	1.83 ± 0.03 (11 ± 1)	12.6
I21A	2238.51	1.9 ± 0.1 (11 ± 1)	12.7
V23A	2252.54	2.0 ± 0.1 (11 ± 1)	12.8
P18A/I21A/V23A	2184.42	2.5 ± 0.4 (9 ± 2)	12.6
P18A/I21A/F22A/V23A	2108.32	1.7 ± 0.1 (12 ± 2)	12.4

^a^ Mutated residues are in bold. ^b^ Uncertainties are fitting errors to the exponential curve of *I*/*I*_0_ versus G^2^. The *R*_h_ values of the peptides (within the parentheses) were obtained from comparison with the *R*_h_ of dioxane (2.12 Å) [42]. ^c^ Calculated from the scale law: *R*_h_ = (0.027 ± 0.01) MW^(0.50 ± 0.01)^ [43].

**Table 4:** Affinities of the peptides for EIN^sc^ and Rsd^ec^ as measured by ITC and fluorescence.

Peptide ^a^	EIN^sc^		Rsd^ec^	
	*K*_d_ ^a^ (μM)	Δ*H* (kcal/ mol)	*K*_d_ ^a^ (μM)	Δ*H* (kcal/mol)
Wild-type	2.3 ± 0.4 (10 ± 7)	−0.8 ± 0.4	1.4 ± 0.3 (5 ± 2)	1.3 ± 0.4
P18A ^b,c^	(-)	(-)	2.2 ± 0.4 (-)	1.1 ± 0.4
I21A	(-)	(-)	2.6 ± 0.4 (8 ± 1)	1.0 ± 0.4
F22A ^b^	(-)	(-)	5.9 ± 0.7 (11 ± 6)	1.1 ± 0.5
V23A ^c^	9.1 ± 0.9 (9 ± 4)	−6.6 ± 0.5	8.3 ± 0.9 (-)	−0.8 ± 0.5
P18A/I21A/V23A ^b,c^	(-)	(-)	2.6 ± 0.4 (-)	0.6 ± 0.4
P18A/I21A/F22A/V23A	1.1 ± 0.2 (11 ± 6)	0.2 ± 0.4	5.0 ± 0.7 (10 ± 4)	4.3 ± 0.5

^a^ The values within the parentheses for the corresponding protein were obtained from fluorescence titration measurements. Uncertainties indicated for the fluorescence values are fitting errors. ^b^ It was not possible to fit the fluorescence titration curve between the corresponding peptide and EIN^sc^. ^c^ It was not possible to fit the fluorescence titration curve between the corresponding peptide and Rsd^ec^.

**Table 5:** Antibacterial activity of the different HPr peptides against *S. aureus* ^a^.

Peptides	MIC (µM)
Wild-type	40
P18A	120
I21A	>400
F22A	400
V23A	>400
P18A/I21A/V23A	60
P18A/I21A/F22A/V23A	>400

^a^ All experiments were performed in triplicate (sd = 0).

## Data Availability

The data presented in this study are available upon request from the corresponding author.

## Supplementary Materials

The following are available online at https://www.mdpi.com/article/10.3390/ijms221910805/s1.
