# Supplementary material for: Residual Helicity at the Active Site of the Histidine Phosphocarrier, HPr, Modulates Binding Affinity to Its Natural Partners
Source: Int J Mol Sci. 2021 Oct 6;22(19):10805. doi: 10.3390/ijms221910805 (PMC8509676; doi:10.3390/ijms221910805)
Supplement: Supplementary file 1 [file ijms-22-10805-s001.zip › ijms-1399752-supplementary.pdf]

Residual helicity at the active site of the histidine phosphocarrier, HPr, modulates binding affinity to its natural partners

Supplementary Material

José L. Neira, David Ortega-Alarcón, Bruno Rizzuti, Martina Palomino-Schätzlein, Adrián Velázquez-Campoy and Alberto Falcó

Table S1. Chemical shifts (δ, ppm from TSP) of wild-type HPr in aqueous solution (pH 7.2 (50 mM Tris), 10 ºC).

	NH	Hα	Hβ2	Hβ3	Hγ2	Hγ3	Hδ	Hε	Hζ
Gly9	8.36	3.87							
Trp10	8.10	4.59	3.25		10.21 (NH); 7.25 (C2H); 7.48 (C7H); 7.58 (C4H); 7.24 (C5H); 7.14 (C6H)				
Ala11	8.19	4.14	1.20 (Me)						
Glu12	8.15	4.12	1.91; 2.02		2.29				
Gly13	8.37	3.87							
Leu14	8.03	4.21	1.52; 1.41		1.50		0.84 (Me)		
His15	8.49	4.62	3.07; 3.18		7.91 (C2H); 7.00 (C4H)				
Ala16	8.27	4.30	1.34 (Me)						
Arg17	8.51	4.57	1.73		1.60				
Pro18		4.39	1.89; 1.96		2.30		3.60 3.83		
Ala19	8.59	4.30	1.39 (Me)						
Ser20	8.50	4.55	3.80						
Ile21	8.16	4.12	1.75		1.27; 1.12		0.79 (Me)		
Phe22	8.38	4.61	3.01				7.29	7.32	
Val23	8.09	4.00	1.93		0.87 (Me)				
Arg24	8.45	4.22	1.82		1.70		3.19		
Ala25	8.54	4.26	1.37 (Me)						
Ala26	8.50	4.40	1.39 (Me)						
Thr27	8.39	4.36	4.26		1.27 (Me)				
Ala28	8.55	4.24	1.39 (Me)						
Thr29	8.26	4.23	4.29		1.23 (Me)				
Gly30	8.56	3.95							

Table S2. Chemical shifts (δ, ppm from TSP) of F22A in aqueous solution (pH 7.2 (50 mM Tris), 10 ºC).

	**NH**	**H$_\alpha$**	**H$_{\beta 2}$**	**H$_{\beta 3}$**	**H$_{\gamma 2}$**	**H$_{\gamma 3}$**	**H$_\delta$**	**H$_\varepsilon$**	**H$_\zeta$**
Gly9	8.34	3.88							
Trp10	8.09	4.59	3.25		10.23 (NH); 7.26 (C2H); 7.48 (C7H); 7.58 (C4H); 7.25 (C5H); 7.14 (C6H)				
Ala11	8.19	4.15	1.20 (Me)						
Glu12	8.15	4.16	1.94; 2.01		2.25				
Gly13	8.38	3.88							
Leu14	8.04	4.22	1.52; 1.46				0.86 (Me)		
His15	8.25	4.60	3.03; 3.14		7.90 (C2H); 6.99 (C4H)				
Ala16	8.14	4.26	1.31 (Me)						
Arg17	8.43	4.58	1.78		1.69				
Pro18		4.39	1.87; 1.94		2.32		3.59 3.82		
Ala19	8.59	4.26	1.40 (Me)						
Ser20	8.43	4.46	3.86						
Ile21	8.24	4.16	1.77		1.20		0.89 (Me)		
**Ala22**	8.40	4.36	1.40 (Me)				7.29	7.32	
Val23	8.24	4.05	2.04		0.91 (Me)				
Arg24	8.50	4.09	1.84		1.64		3.19		
Ala25	8.49	4.31	1.40 (Me)						
Ala26	8.49	4.32	1.42 (Me)						
Thr27	8.29	4.35	4.26		1.21 (Me)				
Ala28	8.50	4.32	1.40 (Me)						
Thr29	8.16	4.37	4.16		1.21 (Me)				
Gly30	8.50	3.90							

Table S3. Chemical shifts (δ, ppm from TSP) of V23A HPr in aqueous solution (pH 7.2 (50 mM Tris), 10 ºC).

	**NH**	**H$_{\alpha}$**	**H$_{\beta2}$**	**H$_{\beta3}$**	**H$_{\gamma2}$**	**H$_{\gamma3}$**	**H$_{\delta}$**	**H$_{\varepsilon}$**	**H$_{\zeta}$**
Gly9	8.33	3.88							
Trp10	8.09	4.58	3.25		10.23 (NH); 7.27 (C2H); 7.49 (C7H); 7.58 (C4H); 7.24 (C5H); 7.14 (C6H)				
Ala11	8.18	4.11	1.22 (Me)						
Glu12	8.15	4.14	1.94; 2.02		2.27				
Gly13	8.37	3.89							
Leu14	8.04	4.22	1.55; 1.46				0.82 (Me)		
His15	8.26	4.60	3.02; 3.17		7.92 (C2H); 7.00 (C4H)				
Ala16	8.15	4.31	1.36 (Me)						
Arg17	8.44	4.52	1.81		1.67		3.17		
Pro18		4.41	1.92; 2.01		2.30		3.59 3.80		
Ala19	8.59	4.27	1.40 (Me)						
Ser20	8.36	4.39	3.84; 3.90						
Ile21	8.15	4.12	1.75; 1.06 (Me)		1.40		0.75 (Me)		
Phe22	8.23	4.63	2.95; 3.19				7.26	7.34	
**Ala23**	8.17	4.28	1.37 (Me)						
Arg24	8.29	4.28	1.81		1.71		3.19		
Ala25	8.48	4.37	1.43 (Me)						
Ala26	8.48	4.37	1.42 (Me)						
Thr27	8.29	4.36	4.27		1.21 (Me)				
Ala28	8.48	4.28	1.42 (Me)						
Thr29	8.18	4.34	4.29		1.21 (Me)				
Gly30	8.54	3.93							

Table S4. Chemical shifts (δ, ppm from TSP) of I21A HPr in aqueous solution (pH 7.2 (50 mM Tris), 10 ºC).

	**NH**	**H_{α}**	**$H_{\beta2}$**	**$H_{\beta3}$**	**$H_{\gamma2}$**	**$H_{\gamma3}$**	**H_{δ}**	**H_{ε}**	**H_{ζ}**
Gly9	8.33	3.85							
Trp10	8.09	4.59	3.28		10.23 (NH); 7.24 (C2H); 7.47 (C7H); 7.58 (C4H); 7.23 (C5H); 7.11 (C6H)				
Ala11	8.18	4.12	1.20 (Me)						
Glu12	8.15	4.14	1.92; 2.03		2.29				
Gly13	8.37	3.92							
Leu14	8.04	4.23	1.55		1.46		0.83 (Me)		
His15	8.25	4.58	3.01; 3.12		7.92 (C2H); 7.00 (C4H)				
Ala16	8.16	4.32	1.32 (Me)						
Arg17	8.44	4.53	1.78		1.67		3.18	7.22	
Pro18		4.41	1.94; 1.99		2.31		3.58 3.79		
Ala19	8.63	4.27	1.23 (Me)						
Ser20	8.47	4.36	3.82; 3.89						
**Ala21**	8.36	4.23	1.27 (Me)						
Phe22	8.17	4.58	3.03; 3.07				7.23	7.33	
Val23	8.02	3.98	1.97		0.91 (Me)				
Arg24	8.45	4.26	1.78		1.67		3.22	7.26	
Ala25	8.47	4.32	1.45 (Me)						
Ala26	8.48	4.34	1.37 (Me)						
Thr27	8.30	4.40	4.31		1.21 (Me)				
Ala28	8.45	4.32	1.37 (Me)						
Thr29	8.19	4.34	4.27		1.21 (Me)				
Gly30	8.51	3.97							

Table S5. Chemical shifts (δ, ppm from TSP) of P18A HPr in aqueous solution (pH 7.2 (50 mM Tris), 10 ºC).

	**NH**	**H_{α}**	**$H_{\beta 2}$**	**$H_{\beta 3}$**	**$H_{\gamma 2}$**	**$H_{\gamma 3}$**	**H_{δ}**	**H_{ε}**	**H_{ζ}**
Gly9	8.29	3.85							
Trp10	8.13	4.59	3.27		10.23 (NH); 7.26 (C2H); 7.46 (C7H); 7.58 (C4H); 7.23 (C5H); 7.11 (C6H)				
Ala11	8.20	4.10	1.23 (Me)						
Glu12	8.18	4.14	1.96; 2.03		2.28				
Gly13	8.36	3.92							
Leu14	8.03	4.18	1.56		1.48		0.84 (Me)		
His15	8.28	4.59	3.06; 3.14		7.95 (C2H); 7.02 (C4H)				
Ala16	8.16	4.32	1.35 (Me)						
Arg17	8.39	4.26	1.80		1.63		3.19	7.24	
**Ala18**	8.50	4.33	1.42 (Me)						
Ala19	8.48	4.33	1.41 (Me)						
Ser20	8.48	4.42	3.86						
Ile21	8.08	4.16	1.75; 1.11 (Me)		1.33		0.83 (Me)		
Phe22	8.32	4.63	3.06; 3.08				7.22	7.32	
Val23	8.08	4.00	1.94		0.85 (Me)				
Arg24	8.42	4.23	1.80		1.68		3.23	7.25	
Ala25	8.47	4.32	1.45 (Me)						
Ala26	8.48	4.32	1.43 (Me)						
Thr27	8.30	4.40	4.31		1.23 (Me)				
Ala28	8.48	4.32	1.43 (Me)						
Thr29	8.19	4.34	4.26		1.23 (Me)				
Gly30	8.51	3.97							

Table S6. Chemical shifts (δ, ppm from TSP) of P18A/I21A/V23A HPr in aqueous solution (pH 7.2 (50 mM Tris), 10 ºC).

	**NH**	**H$_{\alpha}$**	**H$_{\beta2}$**	**H$_{\beta3}$**	**H$_{\gamma2}$**	**H$_{\gamma3}$**	**H$_{\delta}$**	**H$_{\varepsilon}$**	**H$_{\zeta}$**
Gly9	8.27	3.88							
Trp10	8.13	4.57	3.27		10.24 (NH); 7.24 (C2H); 7.47 (C7H); 7.58 (C4H); 7.23 (C5H); 7.11 (C6H)				
Ala11	8.22	4.10	1.22 (Me)						
Glu12	8.18	4.13	1.94; 2.03		2.28				
Gly13	8.36	3.91							
Leu14	8.02	4.18	1.56		1.47		0.84 (Me)		
His15	8.34	4.56	3.10; 3.18		8.15 (C2H); 7.10 (C4H)				
Ala16	8.12	4.33	1.40 (Me)						
Arg17	8.39	4.26	1.82		1.66		3.16	7.23	
**Ala18**	8.34	4.41	1.39 (Me)						
Ala19	8.40	4.27	1.39 (Me)						
Ser20	8.32	4.33	3.85; 3.93						
**Ala21**	8.32	4.23	1.28 (Me)						
Phe22	8.12	4.55	3.02; 3.19				7.26	7.35	
**Ala23**	8.20	4.26	1.39 (Me)						
Arg24	8.26	4.27	1.80		1.63		3.20	7.23	
Ala25	8.34	4.32	1.39 (Me)						
Ala26	8.40	4.32	1.42 (Me)						
Thr27	8.30	4.40	4.31		1.20 (Me)				
Ala28	8.43	4.37	1.43 (Me)						
Thr29	8.17	4.34	4.26		1.20 (Me)				
Gly30	8.48	3.92							

Table S7. Chemical shifts (δ, ppm from TSP) of P18A/I21A/F22A/V23A HPr in aqueous solution (pH 7.2 (50 mM Tris), 10 ºC).

	NH	H_α	$H_{\beta2}$	$H_{\beta3}$	$H_{\gamma2}$	$H_{\gamma3}$	H_δ	H_ε	H_ζ
Gly9	8.33	3.88							
Trp10	8.14	4.57	3.26		10.23 (NH); 7.24 (C2H); 7.48 (C7H); 7.58 (C4H); 7.26 (C5H); 7.15 (C6H)				
Ala11	8.23	4.11	1.18 (Me)						
Glu12	8.18	4.14	1.95		2.25				
Gly13	8.35	3.88							
Leu14	8.02	4.15	1.53		1.43		0.83 (Me)		
His15	8.31	4.53	3.05; 3.13		8.03 (C2H); 7.07 (C4H)				
Ala16	8.17	4.29	1.39 (Me)						
Arg17	8.37	4.22	1.79		1.65		3.15	7.23	
**Ala18**	8.31	4.35	1.42 (Me)						
Ala19	8.40	4.27	1.42 (Me)						
Ser20	8.32	4.36	3.91; 3.98						
**Ala21**	8.27	4.24	1.21 (Me)						
**Ala22**									
**Ala23**									
Arg24	8.20	4.24	1.80		1.63		3.19	7.23	
Ala25	8.23	4.25	1.44 (Me)						
Ala26	8.38	4.23	1.42 (Me)						
Thr27	8.26	4.27	4.32		1.20 (Me)				
Ala28	8.38	4.38	1.41 (Me)						
Thr29	8.07	4.31	4.27		1.21 (Me)				
Gly30	8.47	3.92							

Table S8. Chemical shifts (δ, ppm from TSP) of wild-type HPr in 40 % TFE (pH 7.2 (50 mM Tris), 10 ºC).

	**NH**	**H$_{\alpha}$**	**H$_{\beta2}$**	**H$_{\beta3}$**	**H$_{\gamma2}$**	**H$_{\gamma3}$**	**H$_{\delta}$**	**H$_{\varepsilon}$**	**H$_{\zeta}$**
Gly9	8.25	3.93							
Trp10	7.79	4.50	3.35		10.01 (NH); 7.29 (C2H); 7.48 (C7H); 7.56 (C4H); 7.22 (C5H); 7.18 (C6H)				
Ala11	7.89	4.09	1.16 (Me)						
Glu12	8.08	4.07	2.03		2.35				
Gly13	8.19	3.87							
Leu14	7.83	4.23	1.67		1.51		0.92 (Me)		
His15	8.03	4.58	3.20; 3.33		7.60 (C2H); 7.01 (C4H)				
Ala16	7.96	4.38	1.46 (Me)						
Arg17	8.09	4.58	2.06		1.60				
Pro18		4.39	2.00; 2.11		2.30		3.73 3.81		
Ala19	8.05	4.05	1.52 (Me)						
Ser20	8.10	4.24	3.70						
Ile21	7.75	3.87	1.91; 1.16 (Me)		1.51		0.83 (Me)		
Phe22	7.67	4.30	3.12; 3.30				7.24	7.28	
Val23	8.12	3.69	2.15		0.95; 1.14 (Me)				
Arg24	8.04	4.00	1.98		1.37				
Ala25	8.28	4.13	1.47 (Me)						
Ala26	8.58	4.08	1.34 (Me)						
Thr27	7.95	4.20	4.37		1.30 (Me)				
Ala28	7.98	4.32	1.52 (Me)						
Thr29	7.91	4.08	4.06		1.34 (Me)				
Gly30	8.09	3.68							

Table S9. Chemical shifts (δ, ppm from TSP) of P18A HPr in 40 % TFE (pH 7.2 (50 mM Tris), 10 ºC).

	**NH**	$\mathbf{H_{\alpha}}$	$\mathbf{H_{\beta2}}$	$\mathbf{H_{\beta3}}$	$\mathbf{H_{\gamma2}}$	$\mathbf{H_{\gamma3}}$	$\mathbf{H_{\delta}}$	$\mathbf{H_{\varepsilon}}$	$\mathbf{H_{\zeta}}$
Gly9	8.26	3.94							
Trp10	7.91	4.52	3.37		9.99 (NH); 7.32 (C2H); 7.50 (C7H); 7.56 (C4H); 7.22 (C5H); 7.18 (C6H)				
Ala11	8.01	4.13	1.17 (Me)						
Glu12	8.38	4.06	2.07		2.34				
Gly13	8.18	3.94							
Leu14	7.85	4.19	1.78; 1.54		1.50		0.93 (Me)		
His15	8.17	4.52	3.28		7.92 (C2H); 7.01 (C4H)				
Ala16	7.91	4.20	1.34 (Me)						
Arg17	8.07	4.06	2.03; 1.85						
**Ala18**	8.52	4.17	1.52 (Me)						
Ala19	7.98	4.18	1.31 (Me)						
Ser20	8.39*	4.15	3.86						
Ile21	7.85	3.77	2.01; 1.17 (Me)		1.79		0.90 (Me)		
Phe22	8.17	4.23	3.28				7.24	7.29	
Val23	8.67	3.62	2.18		0.99; 1.16 (Me)				
Arg24	7.98	4.17	1.98		1.76				
Ala25	8.42	4.12	1.50 (Me)						
Ala26	8.77	4.16	1.26 (Me)						
Thr27	7.99	4.20	4.33		1.32 (Me)				
Ala28	8.08	4.32	1.56 (Me)						
Thr29	7.91	4.08	4.05		1.30 (Me)				
Gly30	8.19	3.92							

Table S10. Chemical shifts (δ, ppm from TSP) of V23A HPr in 40 % TFE (pH 7.2 (50 mM Tris), 10 ºC).

	**NH**	**H_{α}**	**$H_{\beta2}$**	**$H_{\beta3}$**	**$H_{\gamma2}$**	**$H_{\gamma3}$**	**H_{δ}**	**H_{ε}**	**H_{ζ}**
Gly9	8.25	3.89							
Trp10	7.79	4.50	3.36		9.99 (NH); 7.28 (C2H); 7.48 (C7H); 7.56 (C4H); 7.23 (C5H); 7.18 (C6H)				
Ala11	7.90	4.13	1.12 (Me)						
Glu12	8.13	4.07	2.01		2.34				
Gly13	8.18	3.91							
Leu14	7.85	4.18	1.66		1.44		0.84 (Me)		
His15	7.97	4.52	3.11; 3.23		7.83 (C2H); 7.07 (C4H)				
Ala16	7.81	4.30	1.44 (Me)						
Arg17	8.06	4.56	1.94						
Pro18		4.34	1.98; 2.16		2.31		3.77; 3.80		
Ala19	8.05	4.09	1.55 (Me)						
Ser20	8.13	4.53	4.09						
Ile21	7.81	3.84	1.87		1.14; 1.54		0.83 (Me)		
Phe22	7.95	4.32	3.13; 3.26				7.24	7.30	
**Ala23**	8.19	4.09	1.59 (Me)						
Arg24	7.95	4.35	2.00		1.80				
Ala25	8.27	4.12	1.49 (Me)						
Ala26	8.53	4.08	1.35 (Me)						
Thr27	7.94	4.20	4.35		1.32 (Me)				
Ala28	8.05*	4.33*	1.54 (Me)						
Thr29	7.83	4.08	4.06		1.30 (Me)				
Gly30	8.19	3.89							

Table S11. Chemical shifts (δ, ppm from TSP) of I21A HPr in 40 % TFE (pH 7.2 (50 mM Tris), 10 ºC).

	NH	H_{α}	$H_{\beta 2}$	$H_{\beta 3}$	$H_{\gamma 2}$	$H_{\gamma 3}$	H_{δ}	H_{ε}	H_{ζ}
Gly9	8.26	3.94							
Trp10	7.81	4.53	3.35		9.99 (NH); 7.28 (C2H); 7.48 (C7H); 7.56 (C4H); 7.23 (C5H); 7.18 (C6H)				
Ala11	7.90	4.13	1.18 (Me)						
Glu12	8.14	4.07	2.08		2.31				
Gly13	8.20	3.87							
Leu14	7.86	4.23	1.66		1.44		0.90 (Me)		
His15	7.95	4.53	3.09; 3.23		7.95 (C2H); 7.05 (C4H)				
Ala16	7.99	4.35	1.32 (Me)						
Arg17	8.09	4.57	1.94		1.76				
Pro18			2.02; 2.16		2.32		3.73; 3.78		
Ala19		4.12*	1.52* (Me)						
Ser20									
**Ala21**	8.20		1.48 (Me)						
Phe22	7.87	4.31	3.21				7.22	7.27	
Val23	8.10	3.57	2.14		0.98; 1.14 (Me)				
Arg24	8.25	3.92	1.90		1.63				
Ala25	7.94	4.16	1.52 (Me)						
Ala26	8.64	4.01	1.28 (Me)						
Thr27	7.91	4.20	4.36		1.32 (Me)				
Ala28	8.09	4.32	1.52 (Me)						
Thr29	7.90	4.08	4.06		1.32 (Me)				
Gly30	8.10	3.95							

Table S12. Chemical shifts (δ, ppm from TSP) of F22A HPr in 40 % TFE (pH 7.2 (50 mM Tris), 10 ºC)[a].

	NH	Hα	Hβ2	Hβ3	Hγ2	Hγ3	Hδ	Hε	Hζ
Gly9	8.27	3.93							
Trp10	7.82	4.53	3.32		10.03 (NH); 7.28 (C2H); 7.48 (C7H); 7.56 (C4H); 7.23 (C5H); 7.14 (C6H)				
Ala11	7.91	4.13	1.14 (Me)						
Glu12	8.13	4.09	2.02		2.34				
Gly13	8.19	3.92							
Leu14	7.83	4.15	1.55				0.88 (Me)		
His15	7.94	4.56	3.07; 3.19		7.89 (C2H); 7.02 (C4H)				
Ala16	7.92	4.20	1.32 (Me)						
Arg17	8.06	4.57	1.83						
Pro18		4.37	2.01; 2.18		2.35		3.73; 3.79		
Ala19	8.05	4.03	1.53 (Me)						
Ser20	8.04	4.37*	3.77						
Ile21	8.04	3.85	1.99		1.21; 1.64		0.91 (Me)		
**Ala22**	8.08	4.19	1.48 (Me)						
Val23	7.83	3.78	2.03		0.96; 1.08 (Me)				
Arg24	8.07	4.03	2.02						
Ala25	8.36	4.19	1.54 (Me)						
Ala26	8.52	4.16	1.51 (Me)						
Thr27	8.04	4.20	4.37		1.29 (Me)				
Ala28	7.98*	4.32*	1.52 (Me)						
Thr29	7.91	4.15	4.20		1.35 (Me)				
Gly30	8.06	3.85							

[a] The * indicates those residues whose resonances could not be unambiguously assigned.

Table S13. Chemical shifts (δ, ppm from TSP) of P18A/I21A/V23A HPr in 40 % TFE (pH 7.2 (50 mM Tris), 10 ºC)[a].

	**NH**	**H_{α}**	**$H_{\beta2}$**	**$H_{\beta3}$**	**$H_{\gamma2}$**	**$H_{\gamma3}$**	**H_{δ}**	**H_{ε}**	**H_{ζ}**
Gly9	8.31	3.91							
Trp10	7.91	4.51	3.35		9.99 (NH); 7.31 (C2H); 7.49 (C7H); 7.55 (C4H); 7.23 (C5H); 7.14 (C6H)				
Ala11	8.00	4.03	1.16 (Me)						
Glu12	8.35	4.07	2.01		2.37				
Gly13	8.18	3.88							
Leu14	7.86	4.15	1.70		1.52		0.89 (Me)		
His15	8.23	4.51	3.29		8.12 (C2H); 7.11 (C4H)				
Ala16	8.29	4.12	1.56 (Me)						
Arg17	8.38	4.09	2.01						
**Ala18**	8.57	4.16	1.47 (Me)						
Ala19	8.02	4.15	1.49 (Me)						
Ser20	8.26*	4.30	3.93						
**Ala21**	8.39	4.14	1.28 (Me)						
Phe22	8.41	4.30	3.22				7.25	7.29	
**Ala23**	8.26	4.13	1.52 (Me)						
Arg24	8.12	4.03	1.99		1.63				
Ala25	8.00	4.14	1.49 (Me)						
Ala26	8.62	4.03	1.28 (Me)						
Thr27	7.85	4.36	4.18		1.29 (Me)				
Ala28	7.95	4.32	1.52 (Me)						
Thr29	7.90*	4.08	4.06		1.32 (Me)				
Gly30	8.10	3.95							

[a] The * indicates those residues whose resonances could not be unambiguously assigned.

Table S14. Chemical shifts (δ, ppm from TSP) of P18A/I21A/F22A/V23A HPr in 40 % TFE (pH 7.2 (50 mM Tris), 10 ºC)[a].

	**NH**	**Hα**	**Hβ2**	**Hβ3**	**Hγ2**	**Hγ3**	**Hδ**	**Hε**	**Hζ**
Gly9	8.30	3.96							
Trp10	7.91	4.52	3.33		9.99 (NH); 7.31 (C2H); 7.50 (C7H); 7.56 (C4H); 7.19 (C5H); 7.14 (C6H)				
Ala11	8.01	3.99	1.16 (Me)						
Glu12	8.37	4.03	2.05		2.38				
Gly13	8.18	3.91							
Leu14	7.97	4.12	1.72		1.52		0.89 (Me)		
His15	8.22	4.52	3.24		7.95(C2H); 7.06 (C4H)				
Ala16	8.26	4.12	1.52 (Me)						
Arg17	8.40	4.12	2.01						
**Ala18**	8.62	4.12	1.51 (Me)						
Ala19	8.05	4.15	1.53 (Me)						
Ser20	8.26		3.89						
**Ala21**	8.40	4.12	1.49 (Me)						
**Ala22**	8.12	4.15	1.49 (Me)				7.25	7.29	
**Ala23**	8.27	4.17	1.48 (Me)						
Arg24	8.07	4.10	1.98						
Ala25	8.05	4.15	1.51 (Me)						
Ala26	8.62	4.12	1.51 (Me)						
Thr27	7.92	4.39	4.21		1.30 (Me)				
Ala28	8.05	4.34	1.53 (Me)						
Thr29	7.92*	4.40*	4.21*		1.30* (Me)				
Gly30	8.18	3.89							

[a] The * indicates those residues whose resonances could not be unambiguously assigned.

Supplementary figures

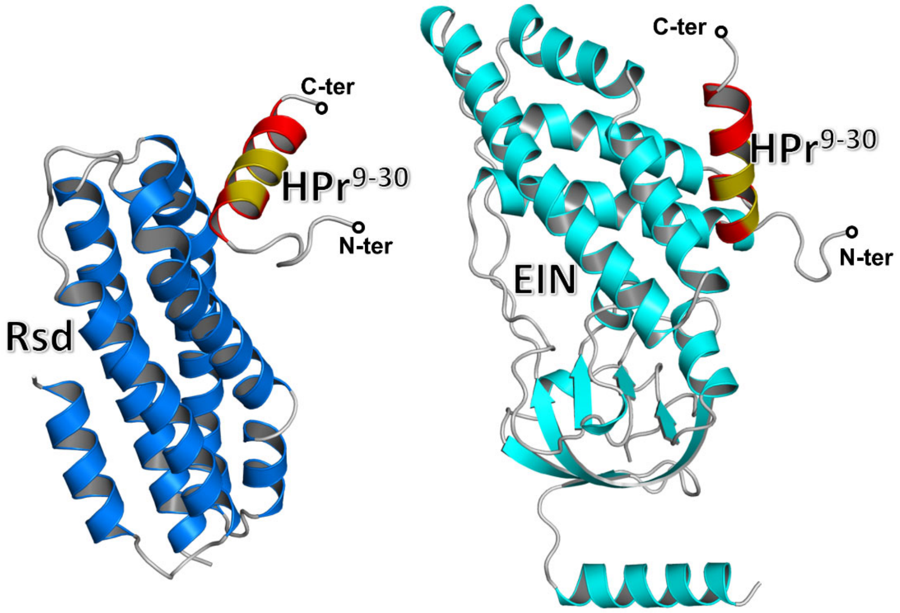

Figure S1. Cartoon representation of (left) Rsd and (right) EIN in interaction with a model of the peptide HPr$^{9-30}$, assumed in helical conformation. The structures are modeled on the basis of the complexes obtained by, respectively, X- ray crystallography with Rsd and NMR spectroscopy with EIN, both bound to intact HPr from E. coli. The termini of the HPr peptide are labeled (N-ter and C-ter), and the residues mutated to Ala are highlighted (yellow).

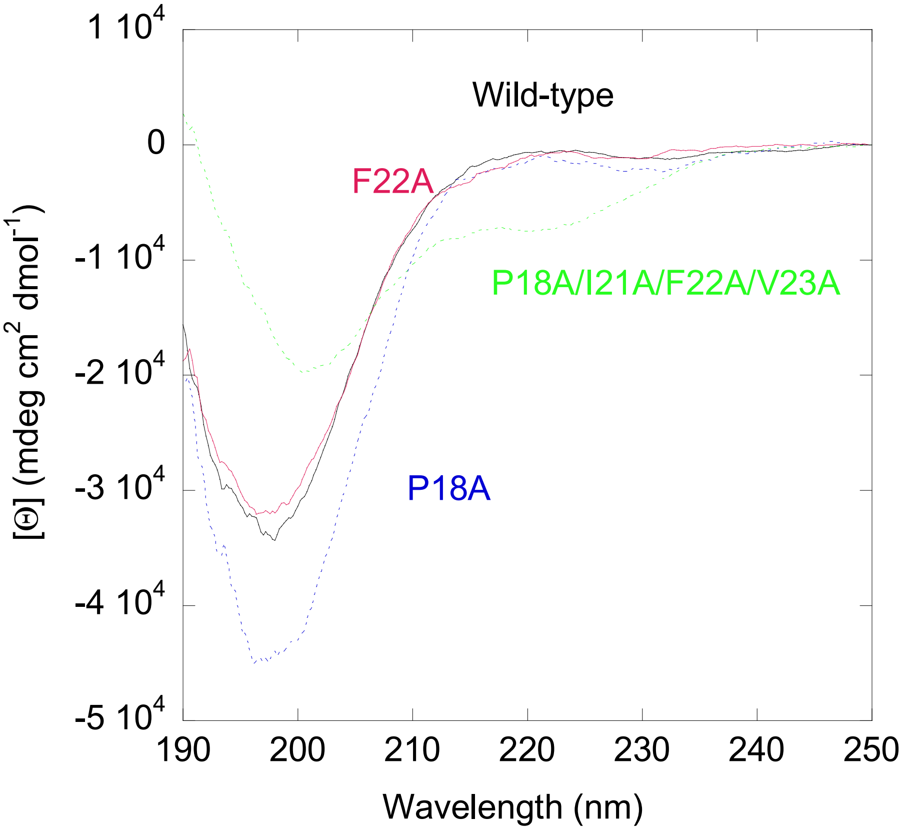

Figure S2. Far-UV CD data of selected isolated peptides in aqueous solution.

FIGURE S3. Conformational characterization of the HPr peptides by NMR in 40 % TFE. NOEs are classified into strong, medium or weak, as represented by the height of the bar underneath the sequence; signal intensity was judged by visual inspection from the NOESY experiments. The dotted lines indicate NOE contacts that could not be unambiguously assigned. The blank squares correspond to the sequential αδ(*i*,*i*+1) NOE observed between a residue preceding a proline and the proline.

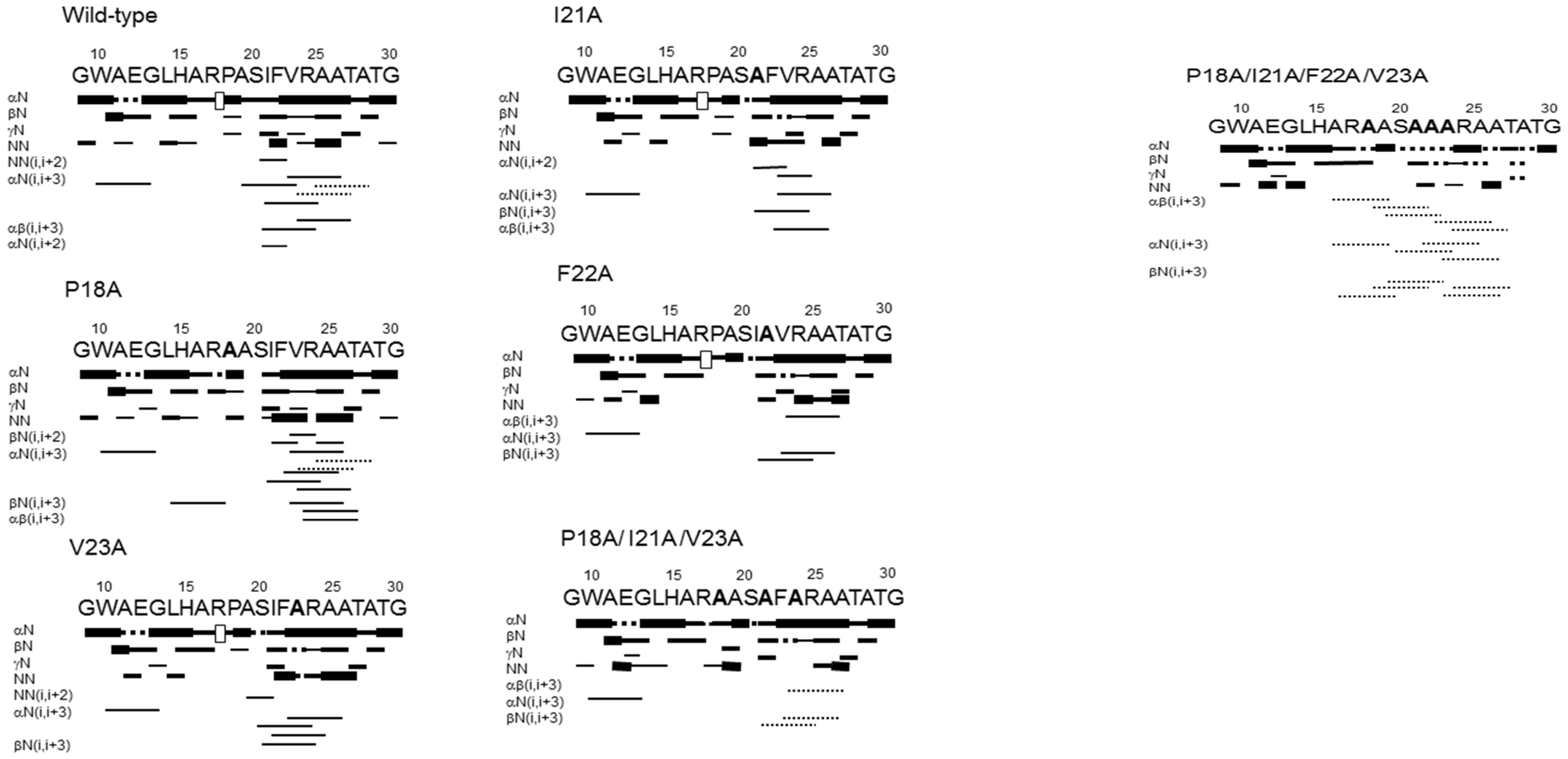

International Journal of
Molecular Sciences

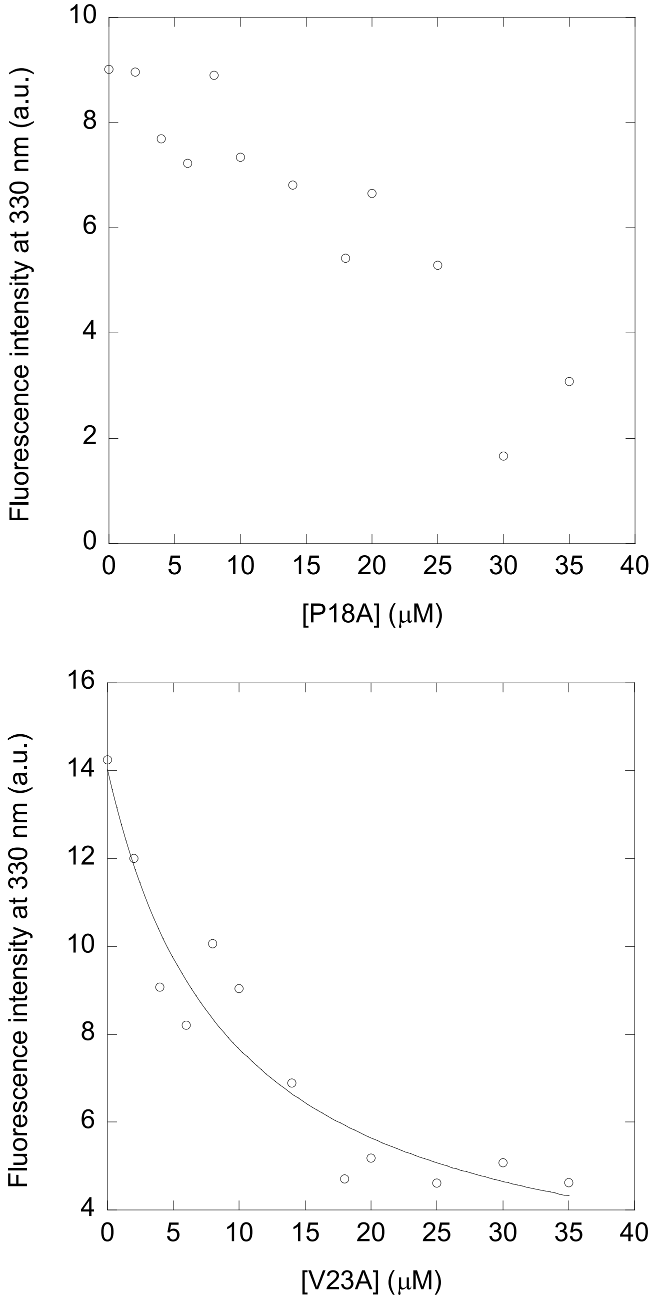

FIGURE S4: Fluorescence titrations of selected peptides. (Top) Titration of P18A onto EIN^{sc}. (Bottom) Titration of V23A onto EIN^{sc}.
